# Isolation, Identification and Evaluation of the Effects of Native Entomopathogenic Fungi from Côte d’Ivoire on *Galleria mellonella*

**DOI:** 10.3390/microorganisms11082104

**Published:** 2023-08-18

**Authors:** Fatoumatou Fofana, Corentin Descombes, Assiri Patrice Kouamé, François Lefort

**Affiliations:** 1 Plants and Pathogens Group, Research Institute Land Nature Environment, Geneva School of Engineering Architecture and Landscape, HES-SO University of Applied Sciences and Arts Western Switzerland, 150 Route de Presinge, 1254 Jussy, Switzerlandcorentin.descombes@hesge.ch (C.D.); 2 Faculty of Natural Sciences, University Nangui Abrogoua, 02 B.P. 801 Abidjan 02, Côte d’Ivoire; kouamass@yahoo.fr

**Keywords:** *Beauveria bassiana*, biological control, *Galleria mellonella*, fall armyworm, *Metarhizium anisopliae*, *Spodoptera frugiperda*

## Abstract

The fall armyworm, *Spodoptera frugiperda* (Lepidoptera: Noctuidae), is a polyphagous pest highly damaging to maize and other food crops in Africa, particularly in Côte d’Ivoire. Chemical pesticides not only have often proved to be unsuccessful, but cause adverse effects on the environment and human health; therefore, entomopathogenic fungi could represent an alternative biocontrol solution. Against this background, fungi were isolated from soil samples collected in maize fields in three regions of Côte d’Ivoire, by the methods of soil dilution and baiting with *Galleria mellonella*. The resulting 86 fungal isolates were phenotypically and genetically identified. The pathogenicity of seven isolates of *Metarhizium* spp., three isolates of *Beauveria bassiana* and two isolates of *Trichoderma* sp. was evaluated on fifth instar larvae (L5) of *G. mellonella*. Larval mortality rates and the median lethal time (LT_50_) were determined seven days after inoculation for each of these selected isolates. The median lethal concentration (LC_50_) was determined for a selection of isolates. *Beauveria bassiana* isolate A214b was the most effective, causing 100% mortality, with an LT_50_ of 2.64 days and an LC_50_ of 1.12 × 10^4^ conidia mL^−1^. Two other promising isolates, A211 and A214a, belonging to *B. bassiana,* caused 100% mortality with LT_50_ values of 3.44 and 4.04 days, respectively. Mortality caused by *Metarhizium* isolates varied from 65.38% to 100%, with *Metarhizium anisopliae* isolate T331 causing 100% mortality with an LT_50_ of 3.08 days at an LC_50_ of 3.33 × 10^4^ conidia mL^−1^. *Trichoderma* sp. isolates were the least pathogenic ones. *Beauveria bassiana* and *Metarhizium* isolates showed to be virulent against the model Lepidopteran *G. mellonella* and will be tested on *S. frugiperda*.

## 1. Introduction

The fall armyworm or American corn moth (*Spodoptera frugiperda* J.E. Smith) is native to tropical and subtropical regions of America. This pest feeds on the leaves and stems of more than 353 plant species [1]. It prefers maize, but is also known as a pest on other crops of economic importance, including cotton, rice, soy, sugar cane, beets, peanut, pumpkin, cabbage, spinach, beans, sorghum, wheat and tomato [2,3]. Moreover, *S. frugiperda* has a very high reproductive rate, considering that a female can lay up to 1000 eggs during its life [4].

This insect attacks all aerial parts of maize, mostly feeding on leaves. They also sometimes cut the stems of seedlings [5]. On young maize plants, whorl damage may affect growing points, preventing cobs’ formation. In severe infestations, plants are completely stripped of their leaves. Feeding deep into the leaf whorl can also destroy developing panicles [1].

In Latin America, this pest has caused production losses of up to 90% [6]. In West Africa it was first reported in 2016 in Nigeria, Benin and Togo, and has subsequently spread to other countries including Côte d’Ivoire [7,8,9]. In August 2016, more than 30,000 ha of maize were destroyed in the regions of northern Benin [5,7].

Chemical control used against this pest proved to be ineffective. In addition, the frequent use of these pesticides causes problems such as the development of resistance in the pest population [10], the reduction of the populations of natural enemies and the pollution of the environment [10]. In view of these disadvantages, it is necessary to find alternative control methods that are effective and respectful of people and the environment. There are currently between 700 and 800 species of fungi listed as entomopathogenic. Most of these fungi belong to the Zygomycetes, Ascomycetes and Chytridiomycetes families [11]. Some entomopathogenic Ascomycetes belonging to the Hypocreales family have a wide spectrum of actions and can infect several species of insects at different stages, thus causing epizootics under natural conditions [12]. Species of the genera *Beauveria*, *Metarhizium*, *Hirsutella*, *Erynia*, *Verticillium*, *Entomophtora*, *Entomophaga* and *Isaria* have already provided efficient fungal isolates, which have been developed as phytosanitary products and commonly used against insect pests in the world. Several isolates belonging to three different genera (*Metarhizium*, *Beauveria* and *Isaria*) were tested in vitro to determine their efficacy on second instar larvae of *S. frugiperda* [3]. However, there are also fungal genera such as *Trichoderma* or *Penicillium*, which are less studied from an entomopathogenic point of view, but might be considered as well [13,14].

Despite the interest aroused by the use of entomopathogenic fungi, very few studies have been carried out in general on these fungi in Western Africa and particularly in Côte d’Ivoire. This study was carried out to generate data and enrich knowledge in this domain. To achieve this, the presence of entomopathogenic fungi in some maize fields in Côte d’Ivoire was assessed. In addition, a pre-screening was carried out on *Galleria mellonella*, in a Swiss laboratory, where it is not allowed to import *S. frugiperda*. *Galleria mellonella* is easily available and lives long enough to perform such tests. It also seems to be a good insect model for observing the virulence of a microorganism against Lepidoptera [15]. The next step of this study will be to test these fungi, in greenhouses and in the field, on *S. frugiperda* larvae on maize, in Côte d’Ivoire.

## 2. Materials and Methods

### 2.1. Sample Collection

The collection of soil samples was made in six localities of maize production in three regions of Côte d’Ivoire (Agnibilékrou; Ferkessedougou; Gagnoa; Korhogo; Ouangolodougou and Tiassalé). In each locality, 5 fields were prospected and 20 samples of 100 g of soil were taken, using a trowel, from the superficial layer (5–10 cm depth) at the foot of randomly selected maize plants according to the method of Thaochan and Sausa-Ard [16]. To avoid contamination from one sampling point to another, the sampling trowel was cleaned with sodium hypochlorite 5% between samplings. Hands were also disinfected with a hydroalcoholic solution (ethanol 70% *v*/*v*). Soil samples were kept in transparent plastic bags with GPS coordinates of the sampling locations (see Table S1). These samples were brought back to the laboratory and those from the same locality were mixed together to form a composite sample.

### 2.2. Isolation of Fungi

Two methods were used for the isolation of fungi, the soil dilution method [17] and the baiting method, using the Lepidoteran *G*. *mellonella* as a bait, developed by Zimmermann [18] and adapted by Laurent et al. [19].

Serial soil dilution suspensions were prepared from 10^−1^ to 10^−7^ starting from a stock solution obtained after dissolving 1 g of soil in 9 mL of distilled water. Then, 100 μL of the dilutions from 10^−2^ to 10^−7^ were spread on potato glucose agar (PGA, Carl Roth, Arlesheim, Switzerland) in Petri plates in three replicates before all Petri dishes were sealed and incubated at 25 °C in the laboratory. Two days after inoculation, the fungal colonies that appeared were subcultured separately on new PGA dishes in order to obtain pure cultures.

As for the insect baiting method, the composite soil samples were each subdivided into three subsamples of 50 g. Each subsample was placed into sterile 500 mL plastic boxes (Sac O_2_ Microbox, Deinze, Belgium) and moistened with 1 mL sterile distilled water; five fifth-instar (L5) larvae of *G. mellonella* were then added in each box. Two control boxes only containing five larvae were also set in parallel. Then, all trays were stored in an incubator Firlabo Bio Concept (Froilabo, Meyzieu, France) at room temperature (25 °C) in the dark. The trays were turned over every day to assure maximum soil–larvae contact. Then, a daily control of the larvae was carried out for three weeks in order to remove the dead larvae from the soil.

Dead larvae were disinfected by dipping in 70% ethanol for 10 s, then in 1% sodium hypochlorite for 3 min, followed by rinsing twice in sterile distilled water for 1 min. After disinfection, each larva was placed into a 9 cm diameter Petri dish on a 9 cm diameter cellulose filter (Rotilabo^®^, Carl Roth), moistened with 1 mL distilled water. Petri dishes were incubated at 25 °C in the dark. The larvae on which fungal sporulation occurred were then removed with sterile forceps and individually deposited on Petri dishes containing PGA amended with 200 mg/L of ampicillin (Carl Roth) 200 mg/L chlortetracycline (Carl Roth), which were then sealed with Parafilm^TM^ and incubated at 25 °C [19]. Several subcultures were sometimes necessary to yield pure fungal cultures.

### 2.3. Molecular Identification of Isolated Strains

The collected isolates were first sorted and classified by morphotypes; then one or two isolates were selected for species identification and cultured in potato glucose broth (PGB; Carl Roth) for 4 to 7 days, with gentle shaking. DNA was extracted following a published protocol [20]. The PCR amplification of the rDNA internal transcribed spacers (ITS) region was carried out by using the primer pair ITS4/ITS5 [21], in a reaction volume of 25 μL. The reaction mixture was prepared with 16.6 μL of ultrapure water, 5 μL from My Taq Reaction Buffer (Bioline GmbH, Germany), 2.5 µL Primer ITS4/ITS5, 0.4 µL My Taq HS DNA polymerase (1 unit/µL; Bioline GmbH), and 0.5 µL of DNA (50 ng/µL). The amplification was carried out in a BIOMETRA thermocycler (Labgene Scientific SA, Châtel St-Denis, Switzerland) under the following conditions: initial denaturation 95 °C for 1 min; followed by 34 cycle of denaturation 95 °C for 15 s; annealing 56 °C for 10 s; elongation 72 °C for 12 s terminated at 4 °C. Following amplification, the PCR products were submitted to electrophoresis on a 1% agarose gel in TBE buffer 1X (Carl Roth) for 35 min under an electrical voltage of 135 mV and visualized under UV with the U: GENIUS^3^ gel reader (Syngene, Cambridge, UK). Successfully amplified PCR were purified with the kit Wizard^®^ SV Gel and PCR Clean-Up System (Promega AG, Dübendorf, Switzerland) and sent to Microsynth AG (Balgach, Switzerland) for Sanger Sequencing. Sequences were edited with FinchTV v. 1.4 (Geospiza Inc., Denver, CO, USA) and identified by searching the NCBI nucleotide database using BLAST (https://blast.ncbi.nlm.nih.gov/Blast.cgi, accessed on 28 February 2022).

### 2.4. Assessing Pathogenicity of Isolates on Galleria mellonella

Conidia were applied to the larvae following the immersion method [22,23]. A conidial suspension at a concentration of 5 × 10^6^ conidia mL^−1^ was prepared by diluting a stock conidial suspension (5 × 10^8^ conidia mL^−1^) in a solution of 10% phosphate buffered saline (PBS, Carl Roth) and 0.03% Tween 20 (Carl Roth) in sterile distilled water. Then, 30 L5 larvae of *G. mellonella* were soaked in 100 mL of the prepared conidial suspension for 10 s. Thirty other larvae representing the controls were also soaked in 100 mL of the diluting solution. For each modality, 10 treated larvae were placed in 500 mL plastic trays (Sac O_2_ Microbox) and incubated in a climatic chamber (Percival Scientific, Iowa, USA) at 25 °C, 60% relative humidity in the dark for 7 days. No food was provided because the L5 stage of *G. mellonella* is the last larval stage before pupation. Larval mortality was monitored daily. Koch’s postulates were verified by performing re-isolation of the entomopathogenic strains from the dead larvae. For this purpose, remains of *G. mellonella* were sterilized and incubated according to the baiting method. Three isolates (A214b, T331 and T34), identified as best performers in the first test, were selected for determination of LC_50_. At this end, conidia suspensions with concentrations of 5 × 10^3^, 5 × 10^4^, 5 × 10^5^ and 5 × 10^6^ conidia mL^−1^ were prepared and applied to the larvae as described previously.

### 2.5. Statistical Analysis

The percentage of larval mortality was calculated using the formula of Abbott [24]. Data were analyzed using STATISTICA 7.1 software. An analysis of variance with one classification criterion (ANOVA1) made it possible to compare the average mortality rates of the larvae according to the fungal isolates. In the event of a significant difference at the 5% level, Tukey’s HSD test was used to determine the homogeneous groups. In addition, the mean lethal time (LT_50_) and lethal concentration (LC_50_) were determined using the log/probit methods of analysis [25].

## 3. Results

### 3.1. Fungi Isolated

**Figure 1:**
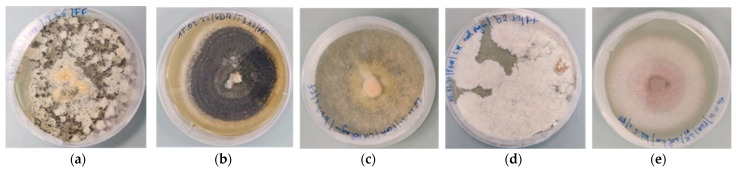
Examples of fungal morphotypes isolated: (**a**) *M*. *anisopliae* isolate T35; (**b**) *Metarhizium* sp. isolate T313; (**c**) *Trichoderma harzianum* isolate A331; (**d**) *B*. *bassiana* isolate A214a; (**e**) *Fusarium oxy-sporum* isolate T251.

**Figure 2:**
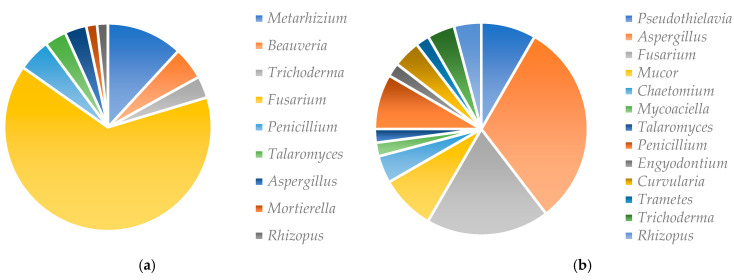
Isolation rates of fungi according to genus and method of isolation. (**a**) Fungi isolated by the baiting method; (**b**) fungi isolated by the soil dilution method.

A total of 86 fungi were isolated (Figure 1): the soil dilution method yielded 27 isolates from 13 genera (Table S2) and 59 isolates belonging to 9 genera were obtained with the baiting method (Table S3). From the baiting method, only one isolate representative of dissimilar individual morphotypes was used for ITS sequencing. The resulting sequences of forty-four isolates were registered in the NCBI Nucleotide database (https://www.ncbi.nlm.nih.gov/nuccore/; accessed on 2 April 2022) under the accessions numbers to ON121940 to ON121983 (Table S4). Figure 2a,b shows the distribution of isolated fungi from both methods.

Fourteen isolates are potentially entomopathogenic according to the literature (Table 1). Twelve of these strains were obtained from the baiting method; that is, five isolates of *Metarhizium* sp., two isolates of *M*. *anisopliae*, three isolates of *B. bassiana* and two isolates of *Trichoderma* sp. The other two isolates of *Trichoderma* sp. were obtained through the soil dilution method.

### 3.2. Pathogenicity of Isolates on Galleria mellonella Larvae

**Figure 3:**
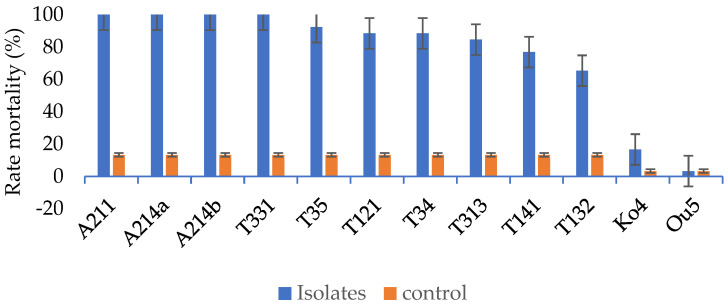
Mortality rate of *G. mellonella* larvae according to isolates, 7 days after inoculation with 5 × 10^6^ conidia mL^−1^.

The results obtained showed that all fungi tested in this study except *Trichoderma* sp. strains were pathogenic against *G. mellonella*. The first dead larvae were observed 2 days after treatment for all isolates of *B*. *bassiana* and for *Metarhizium* spp. isolates (T331, T34, T35). Larval mortality rates varied from 3.33% to 100%. *Beauveria bassiana* isolates (A211, A214a, A214b) and *M*. *anisopliae* isolate T331 induced 100% mortality of *G. mellonella* larvae. *Metarhizium anisopliae* isolate T35 and *Metarhizium* sp. isolates (T34, T121, T313, T141, and T132) caused 93.33%, 90%; 90%; 86.67%; 80% and 70% mortality, respectively. The mortality rates were 16.33% for the *T*. *longibrachiatum* isolate Ko4 and 3.33% for *T*. *asperellum* isolate Ou5 (Figure 3). Statistical analysis showed significant differences in mortality rates between *Metarhizium* spp. and *B*. *bassiana* isolates compared to controls. However, no differences were observed between mortality rates of *Metarhizium* spp. and *B*. *bassiana* isolates. For *Trichoderma* isolates, mortality rates did not significantly differ from the control (F = 17.39; DL = 10; *p* ˂ 0.000).

**Figure 4:**
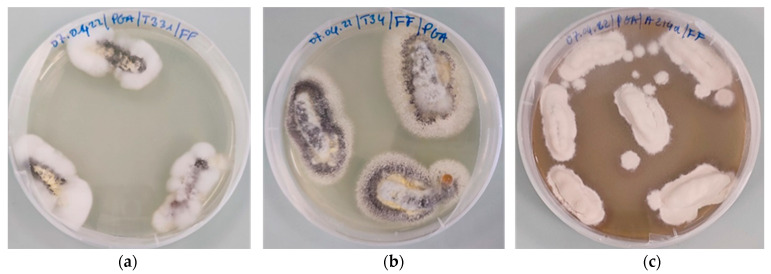
Fungi re-isolation from cadavers on PGA. (**a**) *M*. *anisopliae* isolate T331; (**b**) *Metarhizium* sp. isolate T34; (**c**) *B*. *bassiana* isolate A214a.

Koch’s postulates showed that all tested fungi were responsible for larval death (Figure 4), with the exception of *Trichoderma* isolates (Table 2).

Regarding the mean lethal time (LT_50_), *B*. *bassiana* isolate A214b quickly developed its pathogenicity, with an LT_50_ of 2.64 days followed by *M*. *anisopliae* isolate T331 and *B*. *bassiana* isolate A211. The LT_50_ values of these latter were respectively 3.08 days and 3.44 days. The LT_50_ of *B*. *bassiana* isolate A214a and *Metarhizium* sp. isolates T34, T35, T121, T132, T141 and T313 were respectively 4.01, 3.55, 5.70, 3.56, 6.41, 5.20 and 6.61 days. No *Trichoderma* sp. used in this study yielded a 50% mortality of the *G. mellonella* population. The LC_50_ results showed that the rate of mortality increased with the concentration. The lowest mortality percentage (10%) was recorded for larvae inoculated with *Metarhizium* sp. isolate T34 at a concentration of 5 × 10^3^ conidia mL^−1^. This rate increased when larvae were exposed to concentrations 5 × 10^4^, 5 × 10^5^ and 5 × 10^6^ conidia mL^−1^, with mortality rates of 25%, 71%, 42% and 88.57%, respectively. LC_50_ of 1.12 × 10^4^ and 3.47 × 10^4^ conidia mL^−1^ were obtained for isolates A214b and T331. Isolate T34 had an LC_50_ of 2.19 × 10^5^ conidia mL^−1^ (Table 3).

## 4. Discussion

This study enabled us to isolate a wide range of fungi, regardless of the method used. The method of isolation by dilution made it possible to isolate a greater fungal diversity. The majority of these fungi were saprophytic with the genera *Fusarium* and *Aspergillus* in large numbers, unlike the baiting method. These results corroborate those of Sengul and Sera [26], who isolated fungi with a similar soil dilution method. In their works, the genera *Penicillium*, *Aspergillus*, *Fusarium* and *Trichoderma* were the most abundant. In addition, the genera *Aspergillus*, *Fusarium* and *Paecilomyces* were isolated with the same method by Ibrahim et al. [27]. Although the baiting method allowed the isolation of fewer genera, it was way more effective in terms of the isolation of entomopathogenic fungi. However, the number of *Metarhizium* strains isolated was far greater than for *Beauveria bassiana*. Similar results were obtained by Correa et al. [28]. Our results are also similar to those of Baydar et al. [29], who isolated fungi belonging to the genera *Metarhizium* sp., *Beauveria* sp., *Fusarium* spp., *Aspergillus* spp., *Penicillium* spp. and *Paecilomyces lilacinus*, from Turkish soils using the baiting method. The difference between isolation methods could rely on the fact that the dilution method would promote the rapid development of saprophytic fungi on the PGA medium, which could prevent the development of other fungi. In addition, the amount of soil used in dilution would reduce the chance to isolate useful fungi, considering the small quantity of soil (1 g) used for the dilution. The difference could also be explained by the sensitivity of *G. mellonella* to entomopathogenic fungi. This baiting method would therefore be the best way to select for entomopathogenic fungi. According to Meyling [30], who compared different methods used for isolating entomopathogenic fungi, the dilution method allows opportunistic fungi to proliferate on culture media, whereas the baiting method with *G. mellonella* is a very sensitive detection method for selectively isolating entomopathogenic fungi.

Fungi *M*. *anisopliae* and *B*. *bassiana* have been effective on *G. mellonella* larvae. Similar results were also obtained by Saleh et al. [31], who obtained mortalities of 100% and 98.4%, respectively, with *B. bassiana* and *M. anisopliae*. The same rate of mortality (100%) was obtained [32] on *G. mellonella* larvae inoculated with *M. anisopliae* isolate ARSEF 5520 at concentration of 3.6 × 10^6^ conidia mL^−1^. Entomopathogenic fungi *B. bassiana* and *Metarhizium* sp. are capable of infecting different species belonging to the order Lepidoptera. Indeed, efficacy tests on second instar larvae of *S. frugiperda* using the *B. bassiana* isolate BCMU6 showed a 91.67% mortality rate 12 days after inoculation at a concentration of 10^8^ conidia mL^−1^ [33]. Similar results were obtained with *M. anisopliae* and *B. bassiana* on fourth instar larvae of *Spodoptera litura*. Mortality rates of 100% were achieved with two *B. bassiana* isolates (TA-4; KSH-2) and two *M. anisopliae* isolates (KSH-8, KZ-2) [34].

In the present study, the verification of Koch’s postulates showed that larval mortality was caused by the strains tested, with the exception *Trichoderma* isolates, which could not be recovered from dead larvae. This could be explained by the fact that *Trichoderma* sp. isolates would not be responsible for the death of larvae. The shortest LT_50_ of 2.64 days was obtained with *B*. *bassiana* strain A214b. The isolates *M*. *anisopliae* T331 and *B*. *bassiana* A211 had an LT_50_ of 3.08 days and 3.44 days, respectively. These results are similar to those of Oreste et al. [35], who obtained mean survival times (MST) of 2.2 and 2.3 days using two isolates of *B. bassiana* AL1 and ALB55. The LT_50_ and LC_50_ values showed that some fungi, in particular *B*. *bassiana* isolate A214b, *M*. *anisopliae* isolate T331 and *Metarhizium* sp. isolate T34, are highly virulent against *G. mellonella* larvae. These results could be explained by the viability/concentration of the conidia and the action time of the fungi. According to several authors [25,36], the degree of virulence of entomopathogenic fungi is closely related to the viability of conidia.

## 5. Conclusions

This study isolated entomopathogenic fungi from soil samples collected in maize fields in Côte d’Ivoire. The method of isolation by baiting proved to be the most effective.

All the tested fungi were pathogenic to *G. mellonella* with the exception of two *Trichoderma* isolates, which caused very low mortality rates. Isolated *B*. *bassiana*, *M*. *anisopliae* and *Metarhizium* sp. caused larval mortality rates greater than 50%, and up to 100%. The determination of their LT_50_ and LC_50_ has shown that these fungi have insecticidal potential and virulence against *G. mellonella*. These fungi will therefore be assayed against *S. frugiperda* in vitro, in greenhouse and field tests, with the hope to soon deliver a biological control agent against the fall armyworm.

## Figures and Tables

**Table 1:** Fungal strains belonging to potentially entomopathogenic species used for pathogenicity testing on *Galleria mellonella*.

Fungi	Isolates	Accessions Numbers
*B*. *bassiana*	A211	ON121958
*B. bassiana*	A214a	ON121959
*B. bassiana*	A214b	ON121960
*M*. *anisopliae*	T35	ON121962
*M*. *anisopliae*	T331	ON121963
*Metarhizium* sp.	T141	ON121961
*Metarhizium* sp.	T34	N.A.
*Metarhizium* sp.	T121	N.A.
*Metarhizium* sp.	T132	N.A.
*Metarhizium* sp.	T313	N.A.
*Trichoderma asperellum*	Ou5	ON121957
*Trichoderma longibrachiatum*	Ko4	ON121954

**Table 2:** Re-isolation rate after verification of Koch’s postulate according isolates.

Species	Isolates	UASWS’s Numbers	Number of Larvae Tested	Number of Dead Larvae	Re-Isolation Rates (%)
*B. bassiana*	A211	UASWS2633	30	30	90
*B. bassiana*	A214a	UASWS2634	30	30	100
*B. bassiana*	A214b	UASWS2635	30	30	100
*Metarhizium* sp.	T34	N.A.	30	27	80
*M*. *anisopliae*	T35	UASWS2637	30	28	100
*Metarhizium* sp.	T121	N.A.	30	27	100
*Metarhizium* sp.	T132	N.A.	30	21	100
*Metarhizium* sp.	T141	UASWS2636	30	24	70
*Metarhizium* sp.	T313	N.A.	30	26	90
*M*. *anisopliae*	T331	UASWS2638	30	30	90
*T*. *longibrachiatum*	Ko4	UASWS2629	30	5	0
*T*. *asperellum*	Ou5	UASWS2632	30	3	0

Note: the re-isolation rate is the ratio between the number of individuals sporulating after death and the number of individuals tested.

**Table 3:** Mean lethal concentration (LC_50_) and lethal time (LT_50_) according the best isolates by morphotype.

Fungi	Isolates	LC_50_ (Conidia mL^−1^)	LT_50_ (Days)
*B*. *bassiana*	A214b	1.12 × 10^4^	2.64
*M*. *anisopliae*	T331	3.47 × 10^4^	3.08
*Metarhizium* sp.	T34	2.19 × 10^5^	3.56

## Data Availability

Not applicable.

## Supplementary Materials

The following supporting information can be downloaded at: https://www.mdpi.com/article/10.3390/microorganisms11082104/s1. Table S1: Sampled localities, GPS coordinates and samples constitution; Table S2: Fungal isolates obtained by the dilution method; Table S3: Fungal isolates obtained by the baiting method; Table S4: Genetic identifications of isolates from both methods.
