# Supplementary material for: Isolation, Identification and Evaluation of the Effects of Native Entomopathogenic Fungi from Côte d’Ivoire on Galleria mellonella
Source: Microorganisms. 2023 Aug 18;11(8):2104. doi: 10.3390/microorganisms11082104 (PMC10458300; doi:10.3390/microorganisms11082104)
Supplement: Supplementary file 1 [file microorganisms-11-02104-s001.zip › Table S1 rev.pdf]

Table S1: Sampled localities, GPS coordinates and samples constitution.

**Regions**					
North			South		East
**Localities**					
**Korhogo**	**Ferkessedougou**	**Ouangolodougou**	**Tiassalé**	**Gagnoa**	**Agnibilékrou**
9° 25' 0.0012'' N, 5° 37' 0.0012'' W	9° 35′ 37″ N, 5° 11′ 50″	9° 58 '0" N, 5°9 '0"	5° 53' 54.20" N, 4° 49' 22.55" W	6° 08′ 00″ N, 5° 56′ 00″ W	7° 7.8678′ N, 3° 12.249′ W
Field 1	Field1	Field1	Field1	Field1	Field1
Field2	Field2	Field2	Field2	Field2	Field2
Field3	Field3	Field3	Field3	Field3	Field3
Field4	Field4	Field4	Field4	Field4	Field4
Field5	Field5	Field5	Field5	Field5	Field5
Composite sample 1	Composite sample 2	Composite sample 3	Composite sample 4	Composite sample 5	Composite sample 6
