# Supplementary material for: Isolation, Identification and Evaluation of the Effects of Native Entomopathogenic Fungi from Côte d’Ivoire on Galleria mellonella
Source: Microorganisms. 2023 Aug 18;11(8):2104. doi: 10.3390/microorganisms11082104 (PMC10458300; doi:10.3390/microorganisms11082104)
Supplement: Supplementary file 1 [file microorganisms-11-02104-s001.zip › Table S2 rev.pdf]

Table S2: Fungal isolates obtained by the dilution method

	Fungi	Isolates codes	Localities	Sampled localities coordinates
1	*Pseudothielavia arxii*	Ag1	Agnibilékrou	7°7.8678′N,3°12.249′W
2	*Mycoaciella* sp.	Ag2	Agnibilékrou	7°7.8678′N,3°12.249′W
3	*Chaetomium arcuatum*	Ag3	Agnibilékrou	7°7.8678′N,3°12.249′W
4	*Aspergillus* sp.	Ag4	Agnibilékrou	7°7.8678′N,3°12.249′W
5	*Pseudothielavia arxii*	Ag5	Agnibilékrou	7°7.8678′N,3°12.249′W
6	*Neocosmospora solani*	Agi1	Agnibilékrou	7°7.8678′N,3°12.249′W
7	*Neocosmospora solani*	Agi2	Agnibilékrou	7°7.8678′N,3°12.249′W
8	*Mucor irregularis*	Agi3	Agnibilékrou	7°7.8678′N,3°12.249′W
9	*Neocosmospora solani*	Agi4	Agnibilékrou	7°7.8678′N,3°12.249′W
10	*Mucor irregularis*	Agi5	Agnibilékrou	7°7.8678′N,3°12.249′W
11	*Mucor irregularis*	Agi6	Agnibilékrou	7°7.8678′N,3°12.249′W
12	*Chaetomium arcuatum*	Fe3	Ferkessedougou	9° 35′ 37″ N, 5° 11′ 50″W
13	*Fusarium equiseti*	Fei4	Ferkessedougou	9° 35′ 37″ N, 5° 11′ 50″W
14	*Talaromyces* sp.	Ga3	Gagnoa	6° 08′ 00″N, 5° 56′ 00″W
15	*Penicillium* sp.	Ga4	Gagnoa	6° 08′ 00″N, 5° 56′ 00″W
16	*Curvularia* sp.	Ga7	Gagnoa	6° 08′ 00″N, 5° 56′ 00″W
17	*Mucor indicus*	Ko1	Korhogo	9°25'0.0012''N,5°37'0.0012''W
18	*Trametes polyzona*	Ko2	Korhogo	9°25'0.0012''N,5°37'0.0012''W
19	*Trichoderma longibrachiatum*	Ko4	Korhogo	9°25'0.0012''N,5°37'0.0012''W
20	*Pseudothielavia arxii*	Ou1	Ouangolodougou	9°58'0" N, 5°9'0" W
21	*Rhizopus homothallicus*	Ou2	Ouangolodougou	9°58'0" N, 5°9'0" W
22	*Pseudothielavia arxii*	Ou3	Ouangolodougou	9°58'0" N, 5°9'0" W
23	*Pseudothielavia arxii*	Ou4	Ouangolodougou	9°58'0" N, 5°9'0" W
24	*Trichoderma asperellum*	Ou5	Ouangolodougou	9°58'0" N, 5°9'0" W
25	*Curvularia* sp.	T1	Tiassalé	5°53'54.20"N,4°49'22.55"W
26	*Paraengyodontium album*	T2	Tiassalé	5°53'54.20"N,4°49'22.55"W
27	*Neocosmospora solani*	T3	Tiassalé	5°53'54.20"N,4°49'22.55"W
