# Supplementary material for: Isolation, Identification and Evaluation of the Effects of Native Entomopathogenic Fungi from Côte d’Ivoire on Galleria mellonella
Source: Microorganisms. 2023 Aug 18;11(8):2104. doi: 10.3390/microorganisms11082104 (PMC10458300; doi:10.3390/microorganisms11082104)
Supplement: Supplementary file 1 [file microorganisms-11-02104-s001.zip › Table S3 rev.pdf]

Table S3: Fungal isolates obtained by the baiting method

	**Fungi**	**Isolates codes**	**Localities**	**Sampled localities coordinates**
1	*Beauvaria bassiana*	A211	Agnibilékrou	7°7.8678′N,3°12.249′W
2	*Beauvaria bassiana*	A214a	Agnibilékrou	7°7.8678′N,3°12.249′W
3	*Beauvaria bassiana*	A214b	Agnibilékrou	7°7.8678′N,3°12.249′W
4	*Penicillium* sp.	A131	Agnibilékrou	7°7.8678′N,3°12.249′W
5	*Penicillium expansum*	A321	Agnibilékrou	7°7.8678′N,3°12.249′W
6	*Aspergillus terreus*	A324	Agnibilékrou	7°7.8678′N,3°12.249′W
7	*Neocosmospora solani*	A34	Agnibilékrou	7°7.8678′N,3°12.249′W
8	*Trichoderma harzianum*	A331	Agnibilékrou	7°7.8678′N,3°12.249′W
9	*Talaromyces amestolkiae*	A213	Agnibilékrou	7°7.8678′N,3°12.249′W
10	*Talaromyces* sp.	A212	Agnibilékrou	7°7.8678′N,3°12.249′W
11	*Penicillium* sp.	A242	Agnibilékrou	7°7.8678′N,3°12.249′W
12	*Rhizopus arrhizus*	A122	Agnibilékrou	7°7.8678′N,3°12.249′W
13	*Fusarium* sp.	A323	Agnibilékrou	7°7.8678′N,3°12.249′W
14	*Aspergillus terreus*	A325	Agnibilékrou	7°7.8678′N,3°12.249′W
15	*Neocosmospora solani*	A311	Agnibilékrou	7°7.8678′N,3°12.249′W
16	*Neocosmospora solani*	A241	Agnibilékrou	7°7.8678′N,3°12.249′W
17	*Neocosmospora solani*	A332	Agnibilékrou	7°7.8678′N,3°12.249′W
18	*Fusarium oxysporum*	A322	Agnibilékrou	7°7.8678′N,3°12.249′W
19	*Fusarium* sp.	A121	Agnibilékrou	7°7.8678′N,3°12.249′W
20	*Fusarium* sp.	Fe25	Ferkessedougou	9° 35′ 37″ N, 5° 11′ 50″W
21	*Neocosmospora solani*	Fe332	Ferkessedougou	9° 35′ 37″ N, 5° 11′ 50″W
22	*Neocosmospora solani*	Fe11	Ferkessedougou	9° 35′ 37″ N, 5° 11′ 50″W
23	*Fusarium oxysporum*	Fe152	Ferkessedougou	9° 35′ 37″ N, 5° 11′ 50″W
24	*Fusarium oxysporum*	Fe331	Ferkessedougou	9° 35′ 37″ N, 5° 11′ 50″W
25	*Fusarium* sp.	Fe23	Ferkessedougou	9° 35′ 37″ N, 5° 11′ 50″W
26	*Fusarium* sp.	Fe321	Ferkessedougou	9° 35′ 37″ N, 5° 11′ 50″W

27	*Fusarium oxysporum*	Fe12	Ferkessedougou	9° 35′ 37″ N, 5° 11′ 50″W
28	*Neocosmospora solani*	G151	Gagnoa	6° 08′ 00″N, 5° 56′ 00″W
29	*Neocosmospora solani*	G35	Gagnoa	6° 08′ 00″N, 5° 56′ 00″W
30	*Neocosmospora solani*	G332	Gagnoa	6° 08′ 00″N, 5° 56′ 00″W
31	*Fusarium oxysporum*	G152	Gagnoa	6° 08′ 00″N, 5° 56′ 00″W
32	*Fusarium oxysporum*	G331	Gagnoa	6° 08′ 00″N, 5° 56′ 00″W
33	*Fusarium oxysporum*	G132	Gagnoa	6° 08′ 00″N, 5° 56′ 00″W
34	*Fusarium* sp.	G212	Gagnoa	6° 08′ 00″N, 5° 56′ 00″W
35	*Fusarium* sp.	Ko211	Korhogo	9°25'0.0012''N,5°37'0.0012''W
36	*Neocosmospora solani*	Ko111	Korhogo	9°25'0.0012''N,5°37'0.0012''W
37	*Neocosmospora solani*	Ko223	Korhogo	9°25'0.0012''N,5°37'0.0012''W
38	*Fusarium oxysporum*	Ko142	Korhogo	9°25'0.0012''N,5°37'0.0012''W
39	*Fusarium oxysporum*	Ko114	Korhogo	9°25'0.0012''N,5°37'0.0012''W
40	*Metarhizium* sp.	T34	Tiassalé	5°53'54.20"N,4°49'22.55"W
41	*Metarhizium* sp.	T121	Tiassalé	5°53'54.20"N,4°49'22.55"W
42	*Metarhizium* sp.	T132	Tiassalé	5°53'54.20"N,4°49'22.55"W
43	*Metarhizium* sp.	T141	Tiassalé	5°53'54.20"N,4°49'22.55"W
44	*Metarhizium* sp.	T313	Tiassalé	5°53'54.20"N,4°49'22.55"W
45	*Metarhizium anisopliae*	T35	Tiassalé	5°53'54.20"N,4°49'22.55"W
46	*Metarhizium anisopliae*	T331	Tiassalé	5°53'54.20"N,4°49'22.55"W
47	*Fusarium oxysporum*	T113	Tiassalé	5°53'54.20"N,4°49'22.55"W
48	*Fusarium oxysporum*	T342	Tiassalé	5°53'54.20"N,4°49'22.55"W
49	*Neocosmospora solani*	T25	Tiassalé	5°53'54.20"N,4°49'22.55"W
50	*Neocosmospora solani*	T112	Tiassalé	5°53'54.20"N,4°49'22.55"W
51	*Neocosmospora solani*	T15	Tiassalé	5°53'54.20"N,4°49'22.55"W
52	*Trichoderma harzianum*	T213	Tiassalé	5°53'54.20"N,4°49'22.55"W
53	*Mortierella* sp.	T221	Tiassalé	5°53'54.20"N,4°49'22.55"W
54	*Fusarium oxysporum*	Ou222	Ouangolodougou	9°58'0" N, 5°9'0" W
55	*Neocosmospora solani*	Ou212	Ouangolodougou	9°58'0" N, 5°9'0" W
56	*Fusarium* sp.	Ou241	Ouangolodougou	9°58'0" N, 5°9'0" W

57	*Fusarium* sp.	Ou143	Ouangolodougou	9°58'0" N, 5°9'0" W
58	*Fusarium* sp.	Ou242	Ouangolodougou	9°58'0" N, 5°9'0" W
59	*Fusarium* sp.	Ou232	Ouangolodougou	9°58'0" N, 5°9'0" W
