# Supplementary material for: Isolation, Identification and Evaluation of the Effects of Native Entomopathogenic Fungi from Côte d’Ivoire on Galleria mellonella
Source: Microorganisms. 2023 Aug 18;11(8):2104. doi: 10.3390/microorganisms11082104 (PMC10458300; doi:10.3390/microorganisms11082104)
Supplement: Supplementary file 1 [file microorganisms-11-02104-s001.zip › Table S4 rev.pdf]

Table S4. Genetic identifications of isolates from both methods

Fungi	Isolates	Accessions numbers	Sampled localities coordinates
Aspergillus sp.	Ag4	ON121942	Agnibilékrou
Aspergillus terreus	A324	ON121966	Agnibilékrou
Beauveria bassiana	A211	ON121958	Agnibilékrou
Beauveria bassiana	A214a	ON121959	Agnibilékrou
Beauveria bassiana	A214b	ON121960	Agnibilékrou
Chaetomium arcuatum	Ag3	ON121941	Agnibilékrou
Curvularia sp.	Ga7	ON121951	Gagnoa
Fusarium equiseti	Fei4	ON121948	Ferkessedougou
Neocosmospora solani	Agi1	ON121944	Agnibilékrou
Neocosmospora solani	Agi2	ON121945	Agnibilékrou
Neocosmospora solani	Agi4	ON121946	Agnibilékrou
Neocosmospora solani	A34	ON121973	Agnibilékrou
Neocosmospora solani	G35	ON121974	Gagnoa
Neocosmospora solani	G151	ON121975	Gagnoa
Neocosmospora solani	T15	ON121976	Tiassalé
Neocosmospora solani	T25	ON121977	Tiassalé
Neocosmospora solani	T112	ON121978	Tiassalé
Fusarium oxysporum	G132	ON121979	Gagnoa
Fusarium oxysporum	T342	ON121980	Tiassalé
Fusarium oxysporum	T113	ON121981	Tiassalé
Fusarium sp.	A323	ON121982	Agnibilékrou
Fusarium sp.	G212	ON121983	Gagnoa
Metarhizium anisopliae	T35	ON121962	Tiassalé
Metarhizium anisopliae	T331	ON121963	Tiassalé
Metarhizium sp.	T141	ON121961	Tiassalé
Mortierella sp.	T221	ON121967	Tiassalé
Mucor indicus	Ko1	ON121952	Korhogo
Mucor irregularis	Agi6	ON121947	Agnibilékrou
Penicillium expansum	A321	ON121971	Agnibilékrou
Penicillium sp.	A242	ON121970	Agnibilékrou
Penicillium sp.	A131	ON121972	Agnibilékrou
Penicillium sp.	Ga4	ON121950	Gagnoa
Pseudothielavia arxii	Ag1	ON121940	Agnibilékrou
Pseudothielavia arxii	Ag5	ON121943	Agnibilékrou
Rhizopus arrhizus	A122	ON121965	Agnibilékrou
Rhizopus homothallicus	Ou2	ON121955	Ouangolodougou
Talaromyces amestolkiae	A213	ON121969	Agnibilékrou
Talaromyces sp.	A212	ON121968	Agnibilékrou
Talaromyces sp.	Ga3	ON121949	Gagnoa
Trametes polyzona	Ko2	ON121953	Korhogo
Pseudothielavia arxii	Ou3	ON121956	Ouangolodougou
Trichoderma harzianum	A331	ON121964	Agnibilékrou
Trichoderma asperellum	Ou5	ON121957	Ouangolodougou
Trichoderma longibrachiatum	Ko4	ON121954	Korhogo
